# Evolution of documents related to the influence of physical activity and functional capacity throughout the aging process: a bibliometric review

**DOI:** 10.3389/fphys.2024.1427038

**Published:** 2024-08-02

**Authors:** Carolina A. Cabo, Víctor Hernández-Beltrán, José M. Gamonales, Orlando Fernandes, Mário C. Espada, José A. Parraca

**Affiliations:** ^1^ Departamento de Desporto e Saúde, Escola de Saúde e Desenvolvimento Humano, Universidade de Évora, Évora, Portugal; ^2^ Comprehensive Health Research Centre (CHRC), University of Évora, Évora, Portugal; ^3^ Instituto Politécnico de Setúbal, Escola Superior de Educação, Setúbal, Portugal; ^4^ Sport Physical Activity and Health Research and Innovation Center (SPRINT), Rio Maior, Portugal; ^5^ Training Optimization and Sports Performance Research Group (GOERD), Faculty of Sport Science, University of Extremadura, Cáceres, Spain; ^6^ Faculty of Education and Psychology, University of Extremadura, Badajoz, Spain; ^7^ Programa de Doctorado en Educación y Tecnología, Universidad a Distancia de Madrid, Madrid, Spain; ^8^ Life Quality Research Centre (CIEQV), Setúbal, Portugal; ^9^ CIPER, Faculdade de Motricidade Humana, Universidade de Lisboa, Lisboa, Portugal

**Keywords:** sedentary lifestyle, elderly, trend, co-authorship, journals

## Abstract

Physical inactivity can lead to frailty and negative health outcomes in middle-aged to older adults. Sedentary individuals have double the risk of death compared to those who engage in high levels of physical activity (PA). The advantages of practicing PA in older age are significant, with regular, moderate-intensity activity (150 min per week)being consistently linked with a decreased risk of chronic disease, cognitive decline, and mortality. Therefore, the study aimed to carry out a bibliometric review related to the terms “Physical activity,” “Functional capacity” and “Aging” including all the documents published in the Web of Science Core Collection until 31st December 2023. The sample was made up of 231 studies related to the topic. The results reported that the first document was published in 1994. However, there was no continuity in the publication of the documents till 1998, which was the first year with at least one document published. Considering 1998 as the first year, it is observed an exponential growth of 77.4%, between the oldest (1997–2008) and contemporaneous studies (2008–2023), in which “Geriatric Gerontology” was the Web of Science category with the highest number of documents (n = 59). The journal “Experimental Gerontology” was associated with the largest number of published documents (n = 7), being indexed in Quartil 2. The years 2009 had the highest number of citations (n = 1811), with a total of 7 documents published and 2018 with the higher number of documents (n = 25). These results reported the importance of PA in elderly people, and how it influences the risk of falls, improving the balance and the functional capacity. Thus, it is important to carry out programmes to promote physical activity to this population and reduce the risk of falls and the presence of diseases.

## 1 Introduction

Human beings are prepared to move, but, interestingly, more and more people practice less physical activity (PA). This is related to current lifestyles, which tend to be more sedentary than active (Woessner et al., 2021). Preventing and managing disabilities in the elderly is a significant public health challenge since aging is a risk factor that contributes to functional decline. This process is continuous and irreversible (Wong et al., 2019; Anton et al., 2020).

Physical inactivity is one of the main modifiable risk factors for mortality (Fletcher et al., 2018). Inactivity is linked to weakness and negative health results among middle-aged and older adults. Sedentary individuals are twice as likely to die as those who engage in high levels of PA (Tieland et al., 2018; Ghram et al., 2021).

Exercise and PA are crucial lifestyle choices that support good aging and are regarded as essential treatment approaches for common geriatric syndromes. Attenuating the deterioration in muscle function and cardiorespiratory fitness, retaining functional capacity, and managing chronic disease are among the known clinical benefits of PA (Langhammer et al., 2018; Meredith et al., 2023). The importance of PA and physical exercise nowadays is very important, with varied benefits for improving health, so it is necessary to have a better approach in the evaluation and control of physical exercises to ensure the continuity of our practitioners to the activity, without risk of injuries, withdrawals or even lack of motivation (Warburton et al., 2006). Normally, PA is defined as body movement produced by the contraction of skeletal muscle that increases caloric needs concerning energy expenditure at rest. Physical exercise is defined as a type of PA that is planned, repetitive, and structured that aims to promote improved health and physical condition (Caspersen et al., 1985).

The benefits of PA in old age are vast. Regular, moderate-intensity activity (150 min per week) consistently reduces the risk of chronic disease, cognitive decline, and mortality (McGarrigle and Todd, 2020). Balance and gait impairments significantly affect one’s ability to perform daily activities and increase the risk of falls, leading to a lower quality of life (Rashid et al., 2021). Exercise programs that emphasize improving strength and balance reduce falls in older adults (Claudino et al., 2021; Wiedenmann et al., 2023). In the same way, these programs should be focused on the maintenance and improvement of the functional capacity of elderly people (de Paiva et al., 2021).

Considering the European Union countries, Portugal is the country with the highest percentage of citizens who never performed PA (36%), higher results than the European average (14%) (Alvarez-Lourido et al., 2023). Elderly people do not escape this tendency, because they do not practice enough PA, causing a decrease in flexibility, body balance (Eckstrom et al., 2020), and quality of life (Fiorilli et al., 2022). As we age, a decline in functionality is experienced, but this can be minimized through regular physical exercise (Daley and Spinks, 2000). This serves to improve cognitive and functional functions, in addition to reducing the risk of falls (Buckinx et al., 2021). PA can support quality of life, lessen loneliness, increase embodied joys in later life, improve wellbeing, and enhance social interactions (Gamonales et al., 2021). It is hardly unexpected that PA is encouraged among older adults given the established advantages (Resna et al., 2022).

In this sense, the World Health Organization (WHO) states that there is clear evidence that regular PA produces important benefits for people over 65 years of age. These are usually less active and therefore have illnesses related to inactivity (Milanovic et al., 2013). Mortality rates from cerebrovascular accidents, coronary heart disease, diabetes, and hypertension, among others, are lower among elderly people who are more active than among those who are less active, consequently they have a better quality of life (Palma et al., 2014). Furthermore, psychological consequences such as low self-esteem, anxiety, depression and, as a social consequence, isolation and negative social image, are determining factors for active aging (Liu et al., 2023). The WHO argues that countries can afford aging if governments, international organizations, and civil society implement active aging policies and programs that improve health, participation, and safety (Guthold et al., 2018; Bull et al., 2020).

Bibliometrics is a statistical method used to analyse scientific publications based on indicators of production, impact, and collaborations. It helps researchers identify potential journals, collaborators, and institutions in specific areas of study (Okubo, 1997). In the same way, the use of bibliometric analysis makes it possible to identify gaps in the literature about research topics. In this way, researchers and scientists will know those aspects that are less worked on, and which can be developed broadly to obtain satisfactory results and conclusions for the scientific community (Gámez-Calvo et al., 2024). This method has been used to explore the scientific production of researchers, institutions, and regions in certain fields. Therefore, the purpose of this research is to analyse the current trends in global publications on the impact of PA and functional capacity during the aging process.

## 2 Materials and methods

### 2.1 Design

Considering Montero & León (Montero and León, 2007) proposal, this study fits within the Theoretical research, because the document’s main purpose was to carry out a bibliometric analysis of the literature. A bibliometric review allows to analyse the state of the art of a theme and identifies future research lines (Hernández-Beltrán et al., 2023).

### 2.2 Data extraction

The document search was carried out in the Web of Science (WoS) database, especially, using the WoS Core Collection. This database provides access to a great number of databases, journals, and disciplines (Moral-Muñoz et al., 2020). In the same way, this database was used to reduce the duplicated studies and to standardize the information related to the topic (Herrera & de las Heras-Rosas, 2020), because between 95% and 99% of the studies indexed in the WoS database, are also in another database such as Scopus (Singh et al., 2021). Within the bibliometric analysis, WoS is the most used database (Hernández-Beltrán et al., 2023).

### 2.3 Search strategy

**FIGURE 1:**
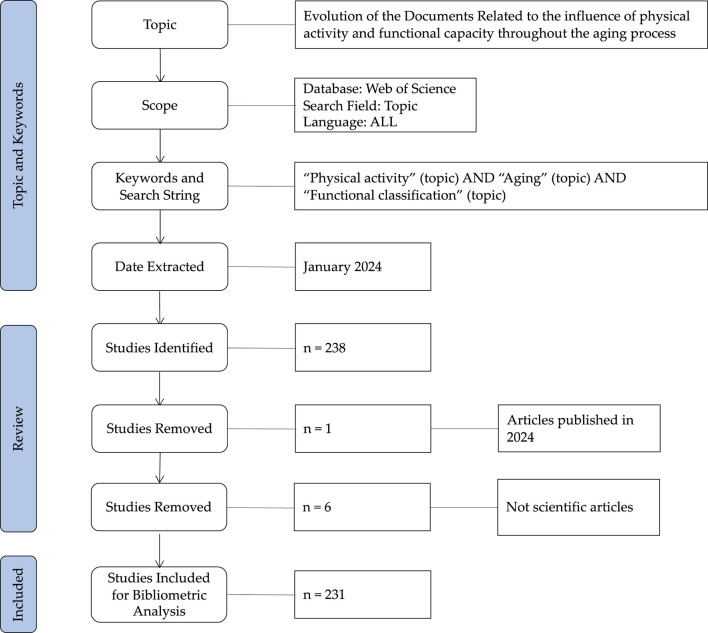
Flow chart of the documents search.

The terms “Physical activity,” “Functional capacity” and “Aging” keywords were used to search the documents. The filter “topic” was used to identify those documents that contain the previous terms in the title, keywords, or abstract of the document, to increase the reliability of the results. Afterward, a total of 238 documents were identified, after applying the inclusion criteria, the sample was reduced to 231 studies for the bibliometric analysis. The documents were published up to 31st December 2023. Firstly, J.M.G. and V.H.B. carried out the search and selection of the documents. Secondly, if there was any disagreement, M.C.E and C.A.C. discussed whether the study was valid and reliable to be selected for the bibliometric analysis. The title and the abstract of the documents were checked to select the most valid documents related to the topic and reduce the risk of bias. Figure 1 shows the process followed in the search of the documents, and the number of studies removed during the phases.

#### 2.3.1 Inclusion criteria

In order to select the most appropriate documents related to the topic, some inclusion criteria were established:

1) The documents must be related to the study of PA and functional capacity of elderly people.
2) To be written in Spanish, English, or Portuguese.
3) The studies must fit such as scientific or review articles (Gamonales et al., 2018).
4) To be published up to 31 December 2023. The documents published in the year 2024 were removed because the year was uncompleted.

### 2.4 Statistical analysis

In order to analyse the sample and the interactions of the authors, organization, countries, and keywords, some bibliometric laws were used. Firstly, De Solla Price’s Law was used to identify the exponential growth of the sample through the R2 coefficient (Price, 1976). The growth dynamics study is an important tool for analyzing the state of the art of a topic and evaluating its trend (Teli and Dutta, 2020). Secondly, the Hirsch Index was calculated to identify the most prominent authors and assess the individual impact of each author (Bihari et al., 2023), using Lotka’s law (Coile, 1977). Finally, to identify the most prominent terms used in each document (Piantadosi, 2014), Zipf’s law was adopted (Zipf, 1932). The key terms are usually used to identify the content of the studies and the trend topics of a research line (Gil-González et al., 2022).

To perform the analysis, data was downloaded in two distinct formats: Excel^®^ (2006 version; Microsoft Corporation, Redmond, WA, United States) and VOSviewer (v. 1.16.19, Center for Science and Technology Studies, Leiden, Netherlands). VOSviewer software was used to carry out the analysis, create, and visualization of the figures. The analysis was accomplished by utilizing the fractionation method, which involved normalization through attraction force (3) and repulsion force (−3).

## 3 Results

### 3.1 Annual publication trends

**FIGURE 2:**
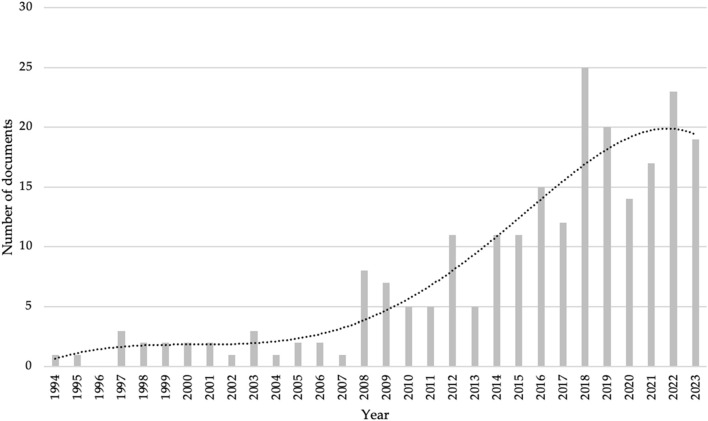
The exponential growth of the sample.

231 documents were selected and included in the analysis. Figure 2 shows the number of documents per year, the x-axis corresponds to the years and the y-axis corresponds to the number of documents, with the oldest articles published in 1994 (Graves et al., 1994) and 1995 (Green and Crouse, 1995). On the other hand, in 1996 years no document was published. Starting from 1998, at least one publication per year has been identified, with a continuity of publication up until the present day. There has been an exponential growth of 77.4% between older studies (1997–2008) and contemporary studies (2008–2023).

### 3.2 WoS categories

It has been observed that within the WoS category, “Geriatrics Gerontology” has the highest number of documents (n = 59). Furthermore, “Experimental Gerontology” is the journal that has published the most documents within this category (n = 7). The data are shown in Table 1.

**TABLE 1:** Documents classified regarding the WOS categories.

WoS categories	Documents	%	Journal name	Documents
Geriatrics gerontology	59	27.44	Experimental gerontology	7
Sport sciences	36	16.74	Journal of aging and physical activity	5
Public environmental occupational health	31	14.41	IJERPH	6
Gerontology	21	9.76	Journal of aging and physical activity	6
Medicine general internal	15	6.97	Preventive medicine	3
Physiology	14	6.51	Frontiers in physiology	3
Nutrition dietetics	13	6.04	Journal of nutrition health aging	3
Rehabilitation	13	6.04	Brazilian journal of physical therapy	3
Cardiac cardiovascular systems	11	5.11	American journal of geriatric cardiology	2
Medicine research experimental	9	4.18	Contemporary clinical trials	3

WoS, web of science. IJERPH, international journal of environmental and public health.

### 3.3 Scientific journals

Table 2 shows the Top 5 most relevant journals related to the study of PA and functional capacity throughout the aging process. Also, it is shown the Quartile and the Rank of the journal regarding the Impact Factor. This result shows the variability of the journals chosen for publishing these studies.

**TABLE 2:** Journals with most published articles related to the study topic.

Journal	Documents	%	IF^a^	Category	Quartile	JIF rank
Experimental gerontology	7	3.24	3.9	Geriatrics and gerontology	Q2	27/54
IJERPH	6	2.77	N/A	Environmental science	N/A	N/A
Journal of aging and physical activity	6	2.77	1.5	Geriatrics and gerontology	Q4	50/54
Archives of gerontology and geriatrics	4	1.85	4.0	Geriatrics and gerontology	Q2	24/54
Plos one	4	1.85	3.7	Multidisciplinary science	Q2	26/73

^a^ Impact factor in the 2022 journal citation reports; JIF, rank, journal impact factor ranking; IJERPH, international journal of environmental and public health.

### 3.4 Most cited documents

To analyse the citations of the documents, first, the H Index of the sample was identified (n = 44). This value allows to know the most potential authors related to the topic (Campos-Soto et al., 2019). H Index is explained as the h number of documents which have at least h citations (Crespo and Simoes, 2019).

**FIGURE 3:**
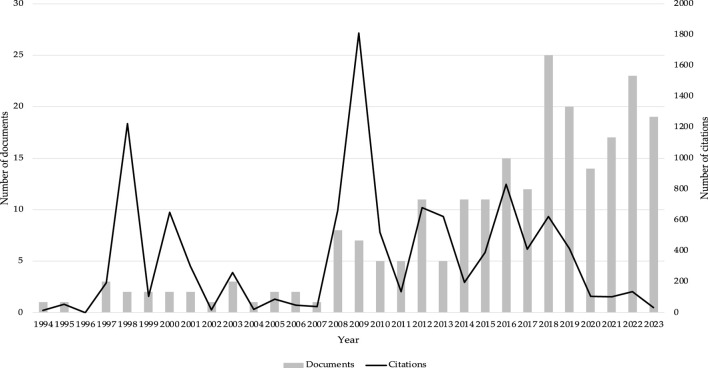
Number of documents and citations per year.

Figure 3 shows the trend in the publication of documents, and the total of citations per year, the x-axis corresponds to the years and the y-axis corresponds to the number of citations. The results reported that 2009, was the year with the highest number of citations (n = 1811), with a total of 7 documents published. On the other hand, 2018 is the year with the largest number of documents (n = 25). It is observed how the number of citations has been so rare from 2020 till now, due to the articles being so recent, with 2022 was the year with the highest number of citations in the last 4 years (n = 137).

Table 3 shows the Top 10 most cited documents related to the topic selected. The results showed that the article written by Chodzko-Zajko et al. (2009) has the highest number of citations. In the same way, this article has the biggest average of citations per year, with a value of 99.5 citations per year. For analyzing the citations of each document, self-citations have been added.

**TABLE 3:** Ten most cited research documents.

Title	Authors	Publication year	Journal title	Total citations	Average citations per year
Exercise and physical activity for older adults	Chodzko-Zajko et al. (2009)	2009	Medicine and science in sports and exercise	1,293	99.5
ACSM position stand: exercise and physical activity for older adults	Mazzeo et al. (1998)	1998	Medicine and science in sports and exercise	1,072	44.7
Social-cognitive and perceived environment influences associated with physical activity in older australians	Booth et al. (2006)	2000	Preventive medicine	508	23.1
Age-related decrease in physical activity and functional fitness among elderly men and women	Milanovic et al. (2013)	2013	Clinical interventions in aging	414	46.0
Updating the evidence for physical activity: summative reviews of the epidemiological evidence, prevalence, and interventions to promote active aging	Bauman et al. (2016)	2016	Gerontologist	404	67.3
Protecting muscle mass and function in older adults during bed rest	English and Paddon-Jones (2010)	2010	Current opinion in clinical nutrition and metabolic care	313	26.1
The danger of weight loss in the elderly	Miller and Wolfe (2008)	2008	Journal of nutrition health and aging	272	19.4
Aging, exercise, and muscle protein metabolism	Koopman and van Loon (2009)	2009	Journal of applied physiology	254	19.5
Physical work capacity in older adults: implications for the aging worker	Kenny et al. (2008)	2008	American journal of industrial medicine	202	14.4
Fish-oil supplementation enhances the effects of strength training in elderly women	Rodacki et al. (2012)	2012	American journal of clinical nutrition	199	19.9

### 3.5 Authors and Co-authors of the documents

**FIGURE 4:**
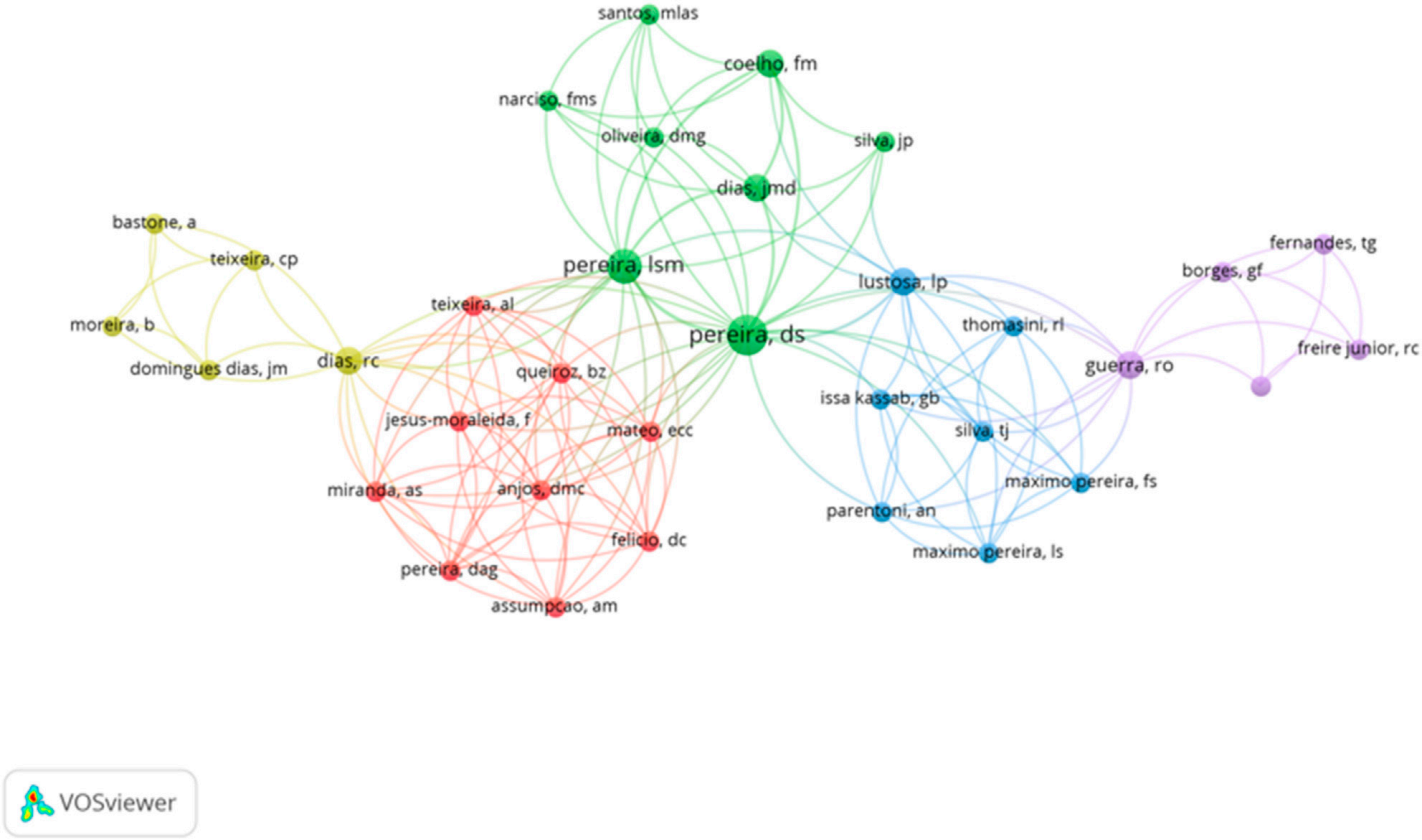
Network regarding the authors and Co-authors.

A total of 1,071 authors were detected, which only eight authors have a minimum of four documents published, and eighty-one authors with two documents. Figure 4 shows the interaction between the authors and co-authors. The author with the highest number of articles published is Aubertin-Leheudre, M., however, he does not appear in the figure because the interactions between the rest of the authors are so rare.

**FIGURE 5:**
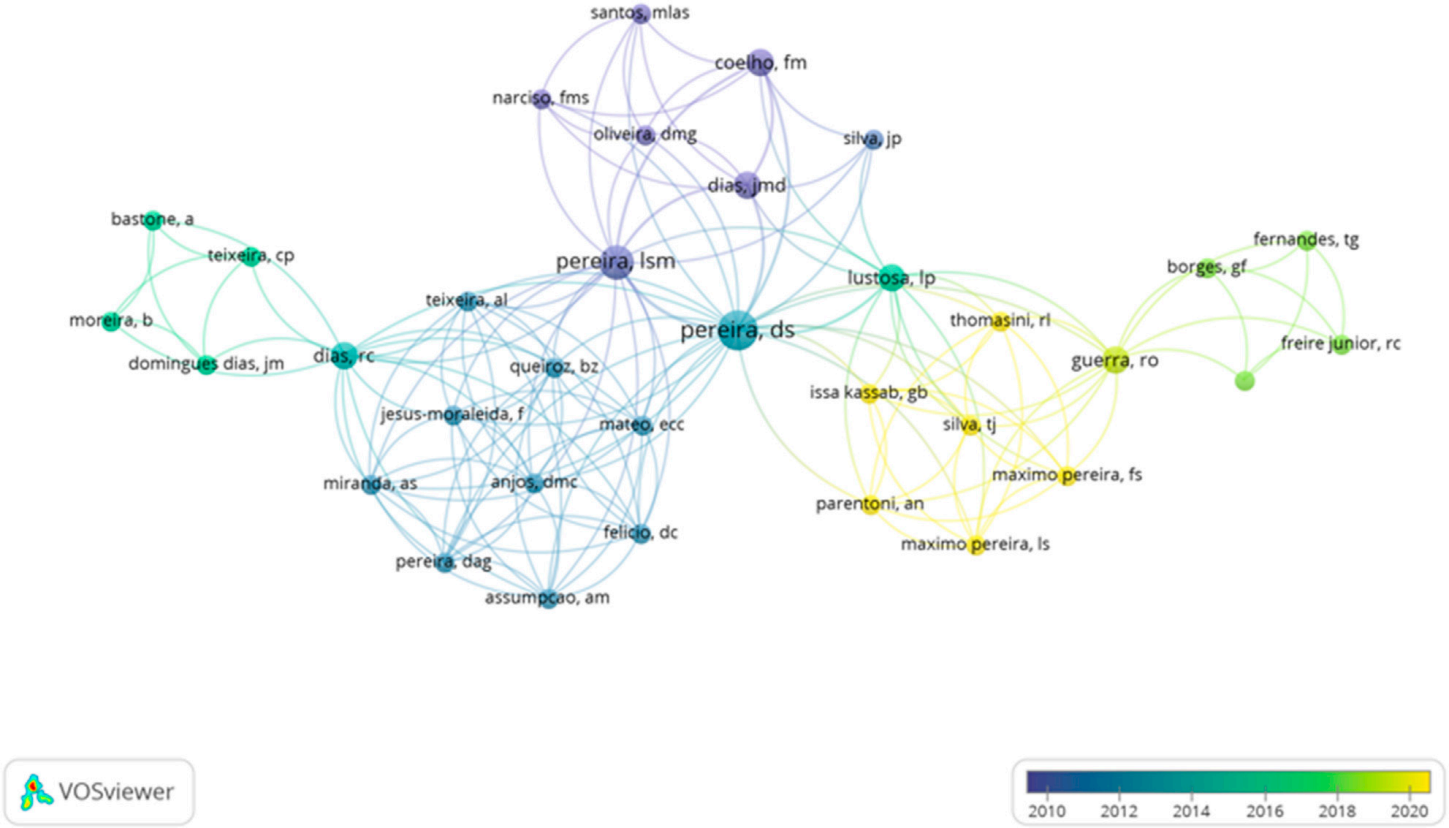
Interactions of the authors considering the timing of publication.

Figure 5 shows the evolution of the group of authors focused on the influence of the PA and functional capacity in elderly people. It shows that the cluster formed by Thomasini, R.L., Issa Kassab, G.B., Silva, T.J., Perentoni, A.N., Maximo Pereira, L.S., and Maximo Pereira, F.S. are the most recent authors which documents are published from 2020 to nowadays. On the other hand, the authors with a higher number of citations show an average of publications between 2010 and 2014.

### 3.6 Publications regarding the countries

**FIGURE 6:**
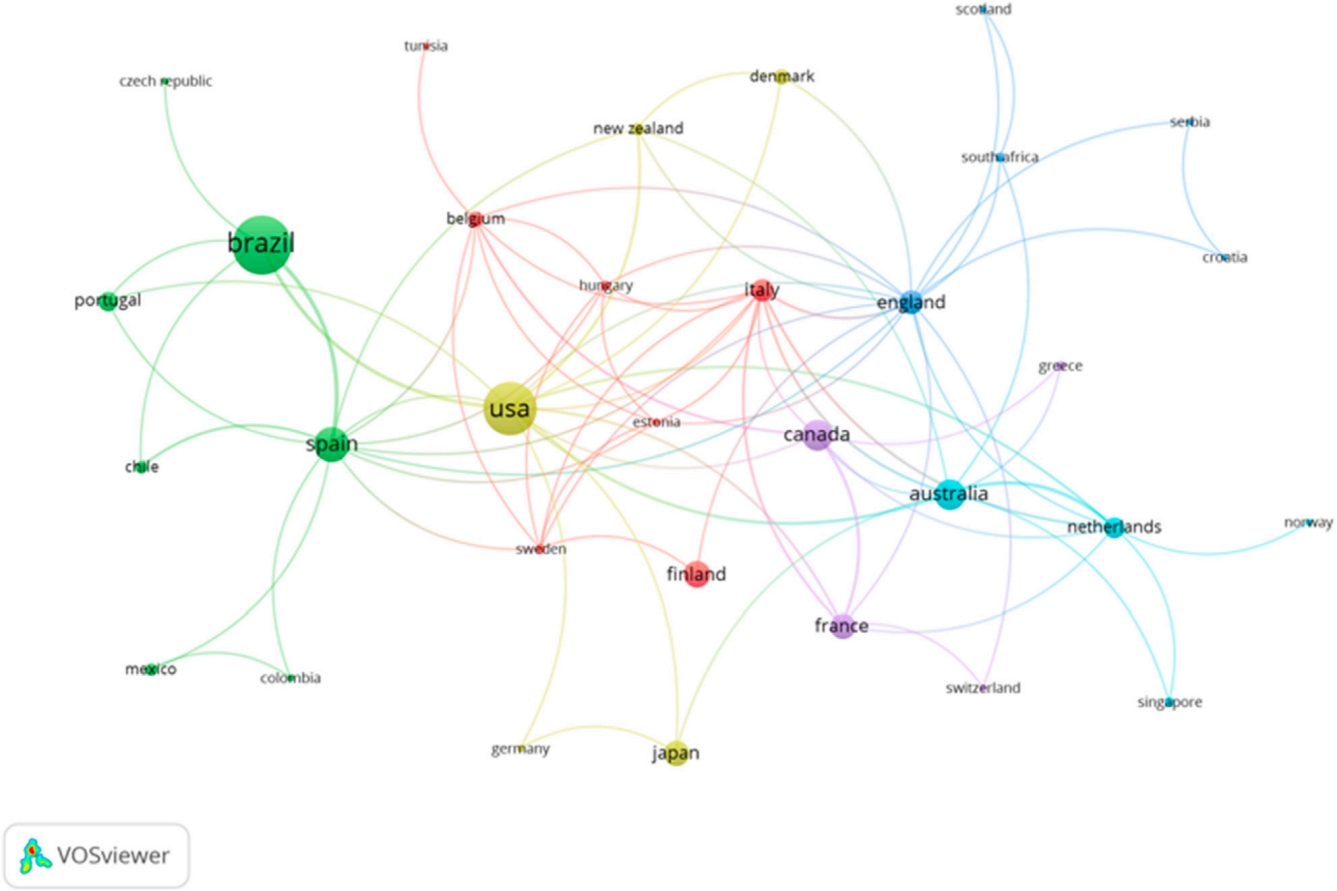
Interactions of the countries based on the number of documents published.

**FIGURE 7:**
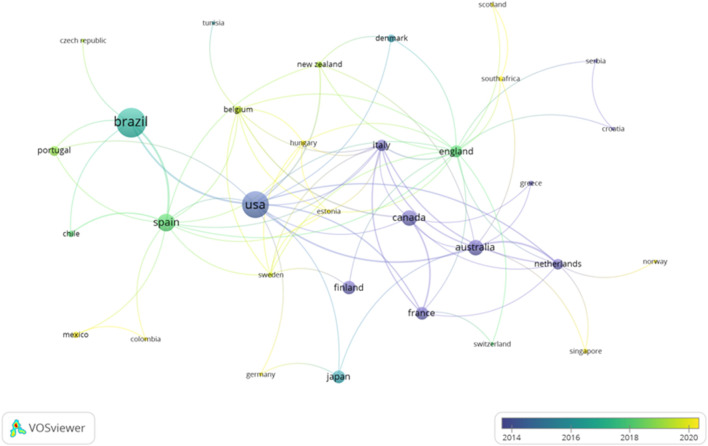
Interactions of the countries in the function of the timing of publication.

Considering the countries which have contributed to the production of the studies, 40 have been identified. Twelve countries had a minimum of 5 documents, and nine countries had a minimum of ten articles. Table 4 shows the top 10 most prolific countries, and the number of papers published. As it is seen Brazil and the United States of America (United States) add up a total of 109 documents. In the analysis, the interaction between the countries is illustrated in Figures 6, 7. The country with the highest number of citations is the United States (n = 2,355), followed by Australia (n = 1,410).

**TABLE 4:** Most prolific countries.

Countries	Documents	%	Citations
Brazil	57	26.51	926
United States	52	24.18	2,355
Spain	21	9.76	434
Australia	16	7.44	1,410
Canada	16	7.44	749
Finland	12	5.58	427
France	11	5.11	360
Japan	11	5.11	187
England	10	4.65	539
Italy	9	4.18	347

USA, United States.

### 3.7 Publications regarding the organisations

**FIGURE 8:**
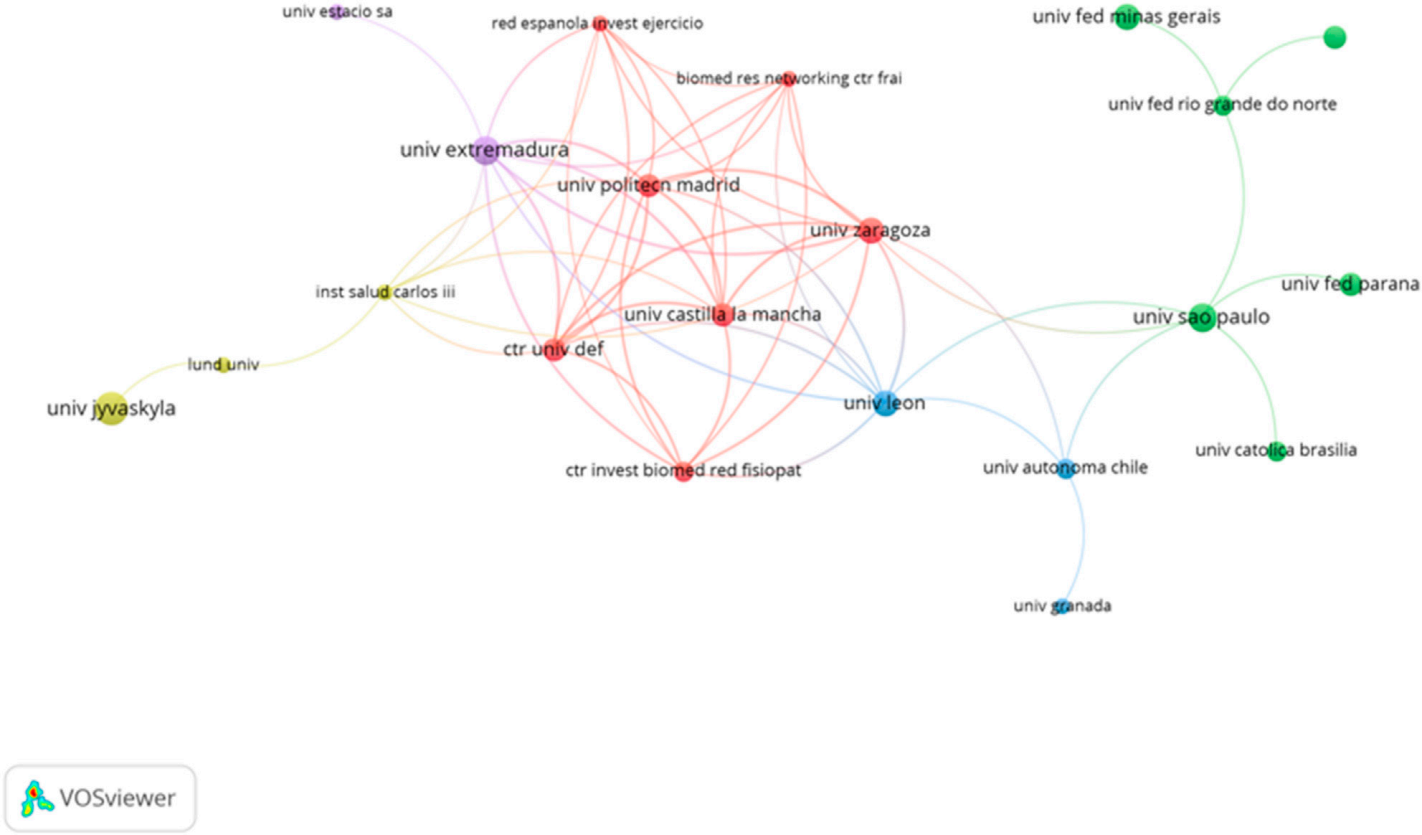
Network regarding the organizations.

Considering the organizations or institutions which participated in the elaboration of the document, a total of 440 were detected. Only eight organizations present a minimum of five documents. In order to identify the relationship between the organizations deeply, only those organizations with a minimum of two documents were included in the analysis (n = 72). “Universidade Federal Do Rio Grande Do Sul,” is the most prolific with a contribution of nine documents. In terms of citations, the University of Sydney is in the first position with a total of 890 citations. Figure 8 shows the interaction between the organizations.

**FIGURE 9:**
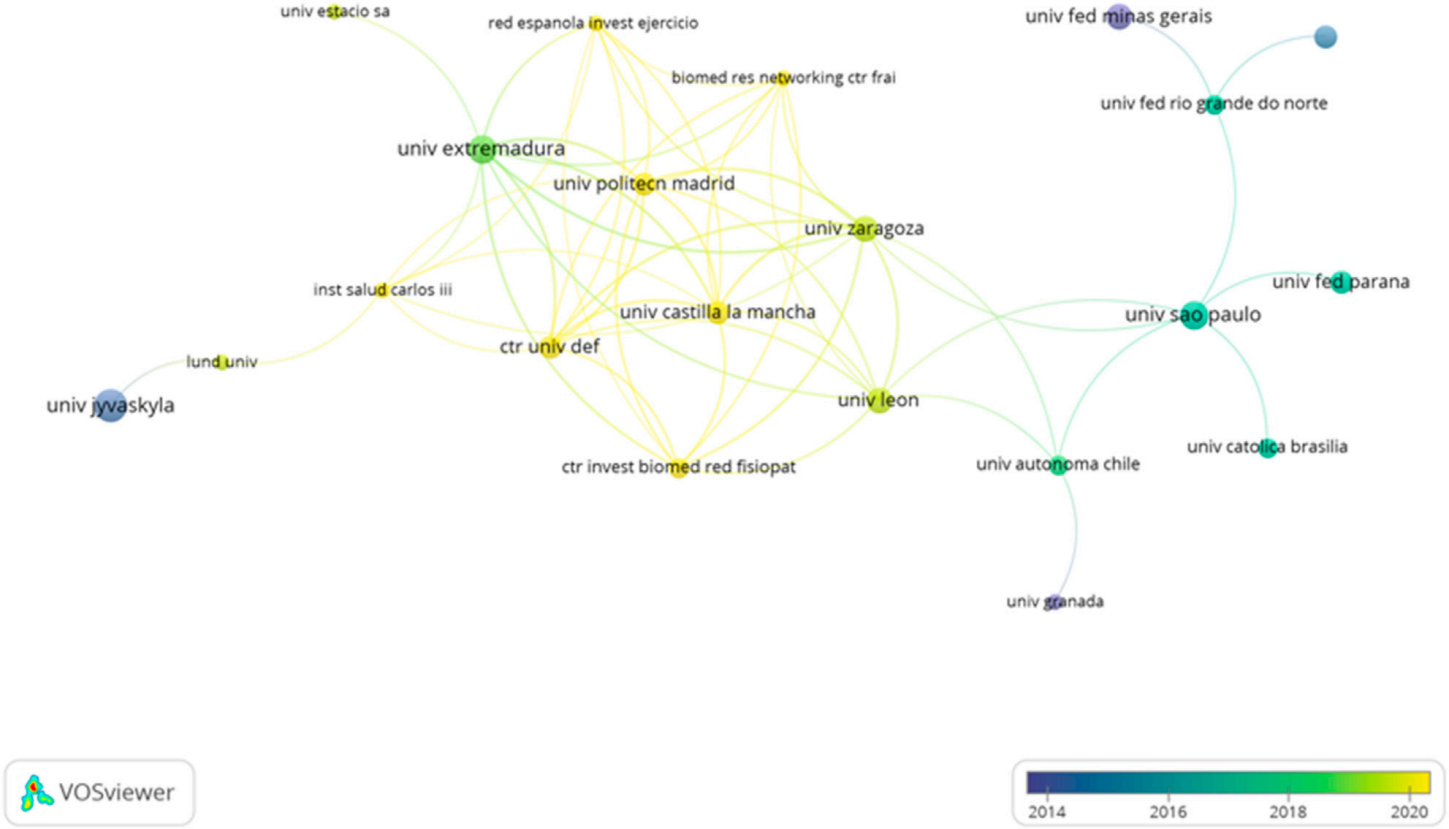
Interactions between the organizations regarding temporalization of publication.

Regarding the timing of publication, it is observed that the “University of Castilla y León” and “Polytechnical University of Madrid” are the most recent organizations considering the average in years of publication. In the same way, the “University of Extremadura” has a recent timing of publication (Figure 9).

### 3.8 Author’s keywords

**FIGURE 10:**
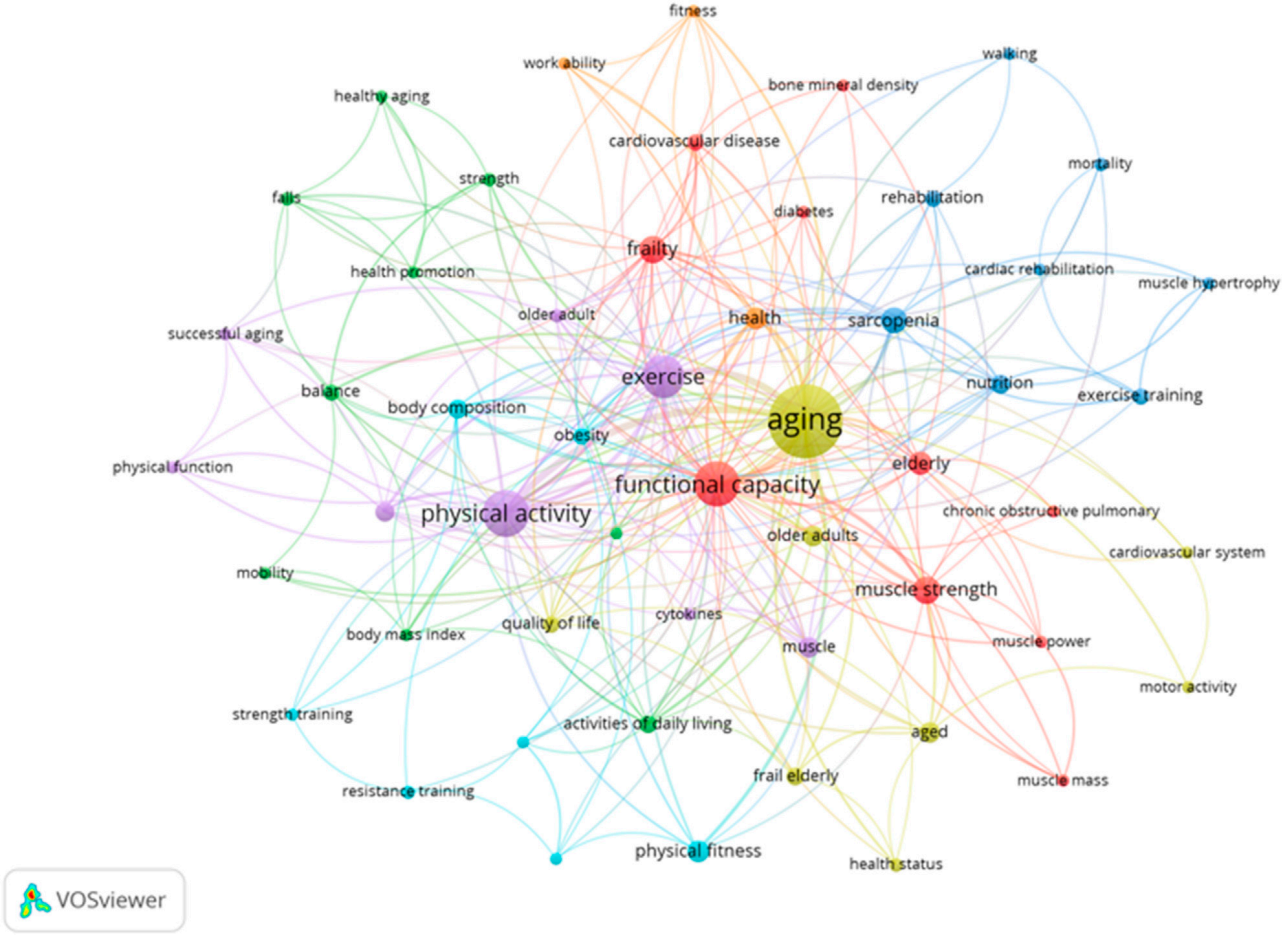
Network of the key terms.

A total of 472 keywords were detected among the studies selected. Only twenty-four words had an equal or higher occurrence than five. Aiming to improve the comprehension and visualization of the interactions, only keywords with a minimum occurrence of three were included (n = 53). The results reported that “aging,” “physical activity,” and “functional capacity,” were the key terms with an occurrence of 102, 42, and 39, respectively (Figure 10).

## 4 Discussion

The aim of the study was to conduct a bibliometric analysis of documents related to the study of the impact of PA on functional capacity during the aging process. The results showed that the first document was published in 1994. However, there was no regular publication of such documents until 1998, which was the first year with at least one document published. If we consider 1998 as the starting year, the number of studies has increased exponentially by 77.4% between older studies (1997–2008) and contemporary studies (2008–2023). The WoS category with the highest number of documents was “Geriatric Gerontology.” Within this category “Experimental Gerontology” was the journal with the largest number of documents published. So, these results reported the importance of PA in elderly people, and how it influences the risk of falls, improving the balance and the functional capacity (Thomas et al., 2019). Thus, it is important to carry out programmes to promote PA to this population and reduce the risk of falls and the presence of diseases.

On the other hand, the article with the highest number of citations is indexed in “Medicine & Science in Sports & Exercise” journal, with a total of 1,293 citations (Chodzko-Zajko et al., 2009). Regarding the total of the documents published related to the topic, an H index of 44 was identified. Thus, this means that there are a minimum of 44 documents with at least 44 citations. The second most cited document was published in 1998 with a total of 1,072 citations, it was also published in the “Medicine & Science in Sports and Exercise” journal. Therefore, it is important to select and publish the document in journals with a high impact factor and use provocative titles (Araujo et al., 2018). In the same way, self-citations play an important role in the increase of the visibility (González-Sala et al., 2019). Self-citations are understood as the number of citations authors make to their own published work. This will increase the number of citations, as well as the visibility of their work, which will allow authors to increase their H-index and 10-index, thereby acquiring greater scientific recognition. These strategies must be followed to increase the visibility of the results and raise the number of citations.

Regarding the authors identified (n = 1,071), only four of them have at least 4 documents published. Aubertin-Leheudre, M., is the author with the highest number of documents carried out (n = 5). However, the results reported that the cluster composed by Thomasini, R.L., Issa Kassab, G.B., Silva, T.J., Perentoni, A.N., Maximo Pereira, L.S., and Maximo Pereira, F.S. are the most recent authors which documents are published from 2020 to now. On the other hand, considering the number of citations, it is observed that the timing of publication fluctuated between 2010 and 2014. Thus, this highlights that most of the publications are so recent because of the interest of the researchers to prevent the risk of falls and improve the quality of life throughout aging. These factors can be improved through PA, and reduce the anxiety, depression (Zhang et al., 2021) and negative social image (Liu et al., 2023). Regular PA prevents chronic diseases, heart problems and the risk of cancer (Haskell et al., 2007). PA can support quality of life, lessen loneliness, in-crease embodied joys in later life, improve wellbeing, and enhance social interactions (Gamonales et al., 2021). It is hardly unexpected that PA is encouraged among older adults given the established advantages (Resna et al., 2022). Therefore, it is recommended to develop future studies focused on strengthening and encouraging PA in this population.

Considering the countries and organizations which collaborated to develop the different studies, a total of 40 countries and 440 organizations were identified. Despite having the highest number of documents (n = 57), Brazil is not the most cited country. The United States holds the record for the largest number of citations (n = 2,355). Additionally, the United States is the most prolific country in various topics studied., such as the analysis of biomechanics in gymnastics (Hernández-Beltrán et al., 2023), the influence of caffeine intake in the fat oxidation process (Gutiérrez-Hellín et al., 2023), or the analysis of the influence of PA in the prevention of breast cancer (Fresno-Alba et al., 2023). On the other hand, considering the organizations, the Universidade Federal Do Rio Grande Do Sul is the most prolific institution with nine documents published. In terms of citations, the University of Sydney is in the first position with a total of 890 citations. “University of Castilla y León” and “Polytechnical University of Madrid” are the most recent organizations considering the timing of publication with a frequency higher than 2020. The timing of publication for the University of Extremadura is recent, but lower compared to the institutions.

Finally, considering the author’s key terms of each study, a total of 472 keywords were identified. “Aging” (n = 102), “physical activity” (n = 42), and “functional capacity” (n = 39), were the terms with the highest occurrence. In order to increase the visibility and impact of the study in future searches, it is important to select the proper keywords (Benito-Peinado et al., 2007). The lack of PA will influence directly the independence of this population, therefore, those seniors who practiced PA regularly (3 or more times per week) will increase their functional capacity and their quality of life (Oliveira et al., 2019). These programmes of PA must be focused on aerobic exercises, to improve muscle strength and functional ability in older adults (Gault and Willems, 2013), as well as walking and manual activities in order to improve fine motor skills (Tomás et al., 2018). Despite the importance of PA, the lack of these interventions produces an increase in sedentary lifestyle in elderly people, thus more strategies must be carried out to prevent and maintain the functional decrease.

This study has certain limitations, such as the number of documents selected, since it assumes the loss of studies that were not indexed in the WoS database, due to the low impact factor of the journal. In this line, when the first search was carried out a total of 238 documents were selected, of which 7 were deleted due to not fixing the inclusion criteria. Finally, the sample was made up of 231 documents, which is a larger number of documents considering the temporal coverage. Similarly, one of the limitations of the study is the selection of key terms. For the correct development of the study, as well as in the extraction of the results, it is necessary to select those words that are closest to the topic and, in this way, eliminate biases in the results. Therefore, in future work, the right planning and selection of keywords should be carried out to reduce the impact of unrelated studies in the final analysis. On the other hand, one of the strengths of the study is that it allows us to know the trends and co-authorship of the documents regarding the country, organization, or authors. In this way, the evolution in the number of publications analyse the influence of PA on the functional capacity of the elderly is known. For future research lines, it is proposed to analyse the current PA programmes and to extract the main findings and results, to obtain a summarized document of the main studies related to PA and its influence on functional capacity. In essence, this research is crucial for its thorough examination and consolidation of literature that demonstrates the significant effect of PA on the aging process. As well as demonstrating the growing interest in this topic in the scientific community. These findings provide guidance for future investigations, policy creation, and the creation of focused interventions aimed at encouraging a healthier and more active lifestyle among elderly populations.

## 5 Conclusion

PA in the elderly allows different benefits to be obtained at various levels, such as the prevention of physical deterioration and the improvement of psychological aspects and social image. PA will improve people’s quality of life, as they will acquire and strengthen movement patterns typical of daily life activities. It will also make them feel better, increasing their personal and social wellbeing. Therefore, keeping a good functional capacity of older people will reduce the risk of disease, cardiovascular problems and sedentary lifestyles Programmes or strategies should be developed to promote PA in this population, through physical work for at least 3 days per week.

This analysis helps to understand the growth and focus areas within the field of study, identifying gaps and opportunities for future research. The findings revealed that the initial document was published in 1994. However, there were no regular publications of such documents until 1998, which marked the first year with at least one document published. If we take 1998 as the starting point, the number of studies has increased exponentially by 77.4% between older studies (1997–2008) and contemporary studies (2008–2023). The category in WoS with the highest number of documents was “Geriatric Gerontology.” Within this category, the journal “Experimental Gerontology” had the largest number of published documents. This points towards the academic and clinical im-portance of this research domain, indicating key platforms for disseminating findings and accessing information relevant to the health and wellness of the aging population.
